# Local recurrence of renal cell carcinoma presented with massive gastrointestinal bleeding: management with renal artery embolization

**DOI:** 10.1186/s42155-019-0054-4

**Published:** 2019-03-15

**Authors:** Donya Farrokh, Masoud Pezeshki Rad, Reihaneh Mortazavi, Reza Akhavan, Bita Abbasi

**Affiliations:** 1 Department of Radiology, Faculty of Medicine, Mashhad University of Medical Sciences, Imam Reza hops, Razi Sq, Mashhad, Iran; 2 Department of Emergency Medicine, Faculty of Medicine, Mashhad University of Medical Sciences, Mashhad, Iran

**Keywords:** Gastrointestinal hemorrhage, Embolization, Therapeutic, Renal cell carcinoma

## Abstract

**Background:**

Gastrointestinal bleeding from renal cell carcinoma metastasis is an uncommon manifestation of tumor recurrence and is usually difficult to control. Palliative trans-catheter embolization to control the bleeding has been used and described in the literature.

**Case presentation:**

The present report describes a 62- years-old male with local recurrence of RCC who presented with upper GI bleeding as the primary manifestation 10 years after right-sided partial nephrectomy. A pseudoaneurysm of renal artery with erosion into the duodenal lumen was responsible for the massive bleeding and was controlled with coil embolization.

**Conclusion:**

This case report highlights the importance of high index suspicion in post-nephrectomy patients for RCC, presenting with new symptoms. Aggressive gastrointestinal workup and adequate awareness of available minimally-invasive endovascular options for controlling GIB in these patients, are of paramount importance.

## Background

Renal cell carcinoma (RCC) is the third most common malignancy of the urinary tract (Zhao et al., 2012; Omranipour et al., 2017). It has a potential of metastasis to almost any organ of the body (Willis, n.d.). Metastasis to the gastrointestinal (GI) tract have been documented in 4–15% of autopsy cases, however, symptomatic GI metastases are rarely encountered in clinical practice (Newmark et al., 1994; Graham, 1947; Fidelman et al., 2010). GI bleeding (GIB) from RCC metastasis is an uncommon manifestation of tumor recurrence and is usually difficult to control (Newmark et al., 1994). Palliative trans-catheter embolization for GIB control has been used and described in the literature (Ohmura et al., 2000). To the best of our knowledge, in all reported cases, GIB was controlled with embolization of celiac, mesenteric, intercostal or lumbar branches that supplied the tumoral tissue (Akhtar et al., 2010).

The present report describes a 62- years-old male with local recurrence of RCC who presented with upper GIB 10 years after right-sided partial nephrectomy. A pseudoaneurysm of renal artery with erosion into the duodenal lumen was responsible for the massive bleeding and was controlled with coil embolization.

## Case presentation

**Fig. 1:**
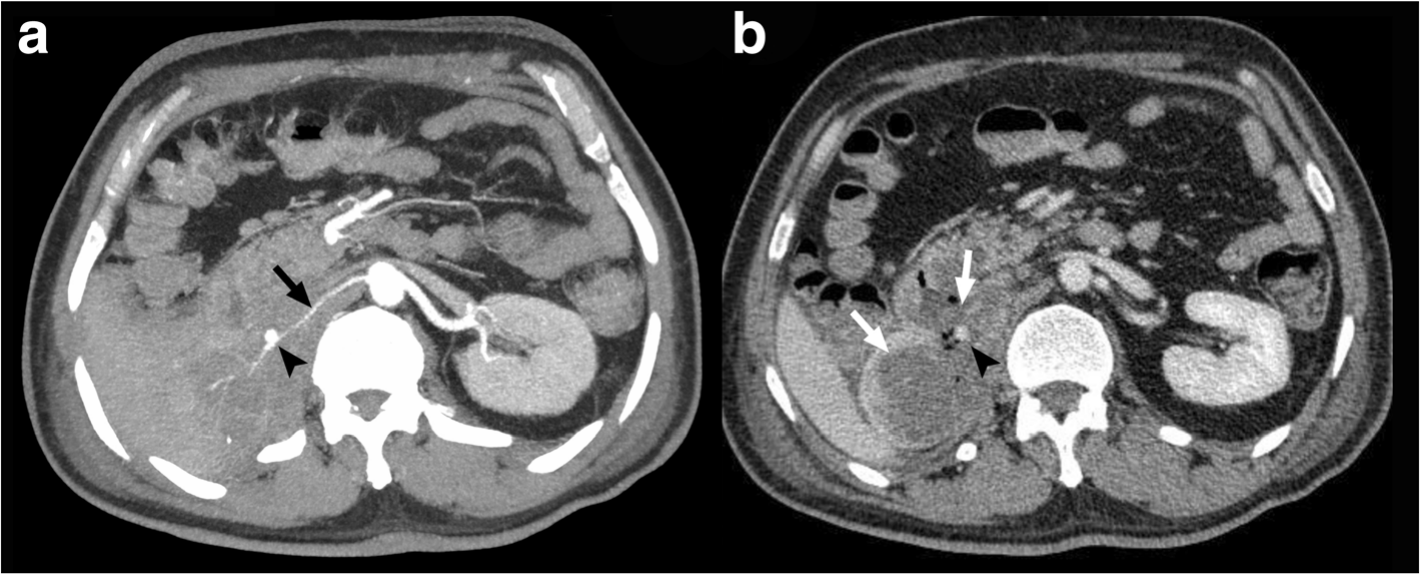
Maximum intensity projection (MIP) reconstruction (**a**) and thin slice (**b**) images of contrast-enhanced abdominal CT scan in the arterial phase show a small right renal artery (arrow in **a**) with a pseudoaneurysm (arrowhead in **a** and **b**), and a hypo-enhancing ill-defined mass originating from the right kidney (asterisk in **b**). There is tumoral erosion and invasion into the adjacent duodenum and remaining renal tissue (white arrows in **b**)

A 62-year-old male presented to the emergency department following an episode of massive GIB. He complained of fatigue, weight loss, and generalized weakness for the last month prior to admission. His past medical history was significant for right renal cell carcinoma (RCC) and partial nephrectomy 10 years ago. Esophagogastroduodenoscopy revealed an irregular, ulcerative mass lesion in the second segment of the duodenum. Contrast enhanced (CT) scan of the abdomen and pelvic showed a heterogeneous soft tissue mass in the nephrectomy bed extending to the second and third segments of the duodenum. There was also a pseudoaneurysm from right renal artery that was located near the soft tissue mass (arrowheads in Fig. 1). The right renal artery was smaller than normal (a finding that was attributed to the long time-interval from previous nephron-sparing surgery), the remaining segment of the right kidney was totally invaded by the recurrent tumor (Fig. 1) and the patient was unstable with active GI bleeding. According to all mentioned facts, we planned an emergency angioembolization of the right renal artery.

**Fig. 2:**
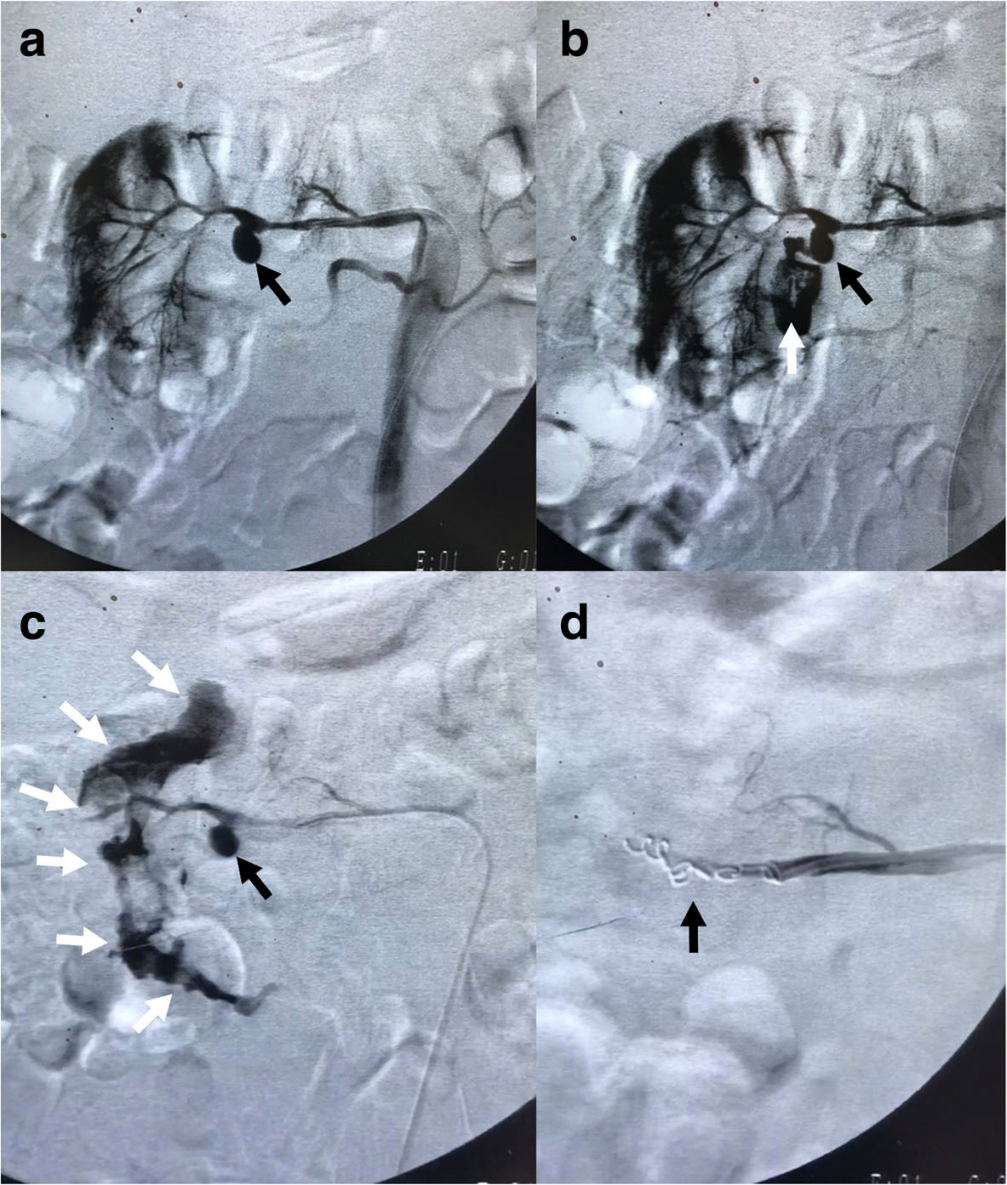
Digital subtraction angiography before (**a**-**c**) and after (**d**) embolization. On the initial angiogram of right renal artery, a pseudoaneurysm is seen (black arrows in **a** to **c**) in the distal extra-renal segment of right renal artery. During contrast injection, rupture and contrast extravasation is noted (white arrow in **b**). The extravasated contrast enters the duodenum and forms the contour of this structure at later phases of injection (white arrows in **c**). Post-embolization angiogram (**d**) shows adequate embolization and exclusion of pseudoaneurysm. The coils are seen in the renal artery (black arrow in **d**)

Angiographic examination of the renal artery revealed a lobulated pseudoaneurysm with intermittent episodes of hemorrhage into the duodenum. The lesion was successfully excluded using coils (Fig. 2) and GIB was controlled.

The patients remained stable after the surgery and was scheduled for systemic chemotherapy. Unfortunately, he decided to leave the hospital against medical advice after two weeks and we could not contact him since then. GI bleeding did not recur during his hospital stay.

## Conclusion

Gastrointestinal metastases are rare causes of GIB (Bhatia et al., 2006). GI involvement can be due to direct extension of primary or recurrent tumor in the renal bed or from hematogenous metastasis (Fidelman et al., 2010). Duodenum is the most common site of GI involvement, given its close proximity to the right kidney (Ouellet et al., 2018). Clinical symptoms of GI involvement are usually related to the tumor-related hemorrhage including melena (Ohmura et al., 2000; Ali et al., 2013), hematemesis (Blake et al., 1995), hematochezia (Ali et al., 2013), haemobilia (Lynch-Nyhan et al., 1987) and iron-deficiency anemia (Kobak et al., 2006), or bowel obstruction (Sadler et al., 2007). Sixty-nine percent of patients with solitary GI metastasis from RCC present with GIB, but massive GIB is a rare occurrence (Ohmura et al., 2000). An ulcerative metastatic mass in the duodenum is the main cause of GIB (Rustagi et al., 2011; Chang et al., 2004; Cherian et al., 2011). Duodenal involvement with pseudoaneurysm formation as the cause of GI bleeding has also been reported in patients with clinical history of RCC in the literature (Blake et al., 1995).

Management of patients with solitary metastatic RCC to the duodenum depends upon the extent and location of duodenal lesion and general conditions of the patient and should be individualized for each patient (Thyavihally et al., 2005). Endoscopic hemostatic control of GIB is usually difficult and in selected cases, intractable GI bleeding can be controlled by endovascular embolization of the tumor-supplying artery (Barth, 1991). This is only a palliative treatment and collateral vessels which have potential for re-bleeding may eventually develop at the site of metastatic lesion. It is also important to keep in mind that small bowel arteries embolization carries a risk of bowel ischemia and infarction (Blake et al., 1995; Lynch-Nyhan et al., 1987). To the best of our knowledge, in all reported cases of GIB secondary to RCC metastasis treated with endovascular approach, a tumor-supplying artery (including mesenteric, celiac, lumbar or intercostal branches) was embolized for hemostatic control. The present case is unique in the way that a pseudoaneurysm from the remaining accessory renal artery (not the tumor-supplying artery) formed a fistulous communication with duodenal lumen and caused massive GI bleeding. Endovascular embolization and coiling of the pseudoaneurysm was performed as an emergent life-saving procedure.

This case report highlights the importance of high index suspicion in post-nephrectomy patients for RCC, presenting with new symptoms. Aggressive gastrointestinal workup and adequate awareness of available minimally-invasive endovascular options for controlling GIB in these patients, are of paramount importance.

## Abbreviations

GI: gastrointestinal
GIB: gastrointestinal bleeding
RCC: renal cell carcinoma
